# Efficient reactive blue 19 decolorization by the comparison of ozonation membrane contacting process and Fenton oxidation†

**DOI:** 10.1039/d1ra01871j

**Published:** 2021-05-17

**Authors:** Anurak Khrueakham, Jidapa Masomboon, Jutamat Roongruang, Sermpong Sairiam

**Affiliations:** Division of Research Administration and Academic Services, Kasetsart University Chalermphrakiat Sakon Nakhon Province Campus Sakon Nakhon 47000 Thailand; Department of Environmental Science, Faculty of Science, Chulalongkorn University Bangkok 10330 Thailand sermpong.s@chula.ac.th; Research Program of Industrial Waste Management – Policies and Practices, Center of Excellence on Hazardous Substance Management (HSM), Chulalongkorn University Bangkok 10330 Thailand; Water Science and Technology for Sustainable Environment Research Group, Chulalongkorn University Bangkok 10330 Thailand

## Abstract

The decolorization of Reactive Blue 19 (RB 19) wastewater by an ozonation membrane contactor and Fenton oxidation was studied. The aims of the study were to investigate the affecting parameters and to compare the performance of RB 19 decolorization by two different processes. The results showed that Fe^2+^ and H_2_O_2_ concentrations for Fenton oxidation and ozone concentration with different membranes for the membrane contacting process played the most important roles in RB 19 decolorization. The optimum conditions for RB 19 decolorization by Fenton oxidation were initial pH 3.0, 1.5 mM H_2_O_2_ and 0.25 mM Fe^2+^; in contrast, the optimum conditions for the membrane contactor were initial pH 11 and 40 mg L^−1^ ozone concentration. Under these conditions, the decolorization of RB 19 by the membrane contactor was almost completed and was higher than by Fenton and photo-Fenton oxidations for 90 min. The decolorizations of RB 19 by Fenton and photo-Fenton oxidations were constant after 30 min, but the decolorization of RB 19 by ozonation with a membrane contactor gradually increased *via* ozone consumption until 90 min operation, which was higher than that of Fenton oxidations. The use of a PVDF-PAM membrane in the membrane contactor resulted in higher decolorization efficiency than a PVDF membrane. The results demonstrated a COD removal efficiency of 63% by an ozonation membrane contacting process using PVDF-PAM, which was lower than that of Fenton oxidation (73%), but resulted in higher BOD_5_/COD and NO_3_^−^ and SO_4_^2−^ releases. Under these conditions, the ozonation membrane contacting process showed the lowest electric energy consumption.

## Abstract 2

The decolorization of Reactive Blue 19 (RB 19) wastewater by an ozonation membrane contactor and Fenton oxidation was studied.

## Graphical abstract

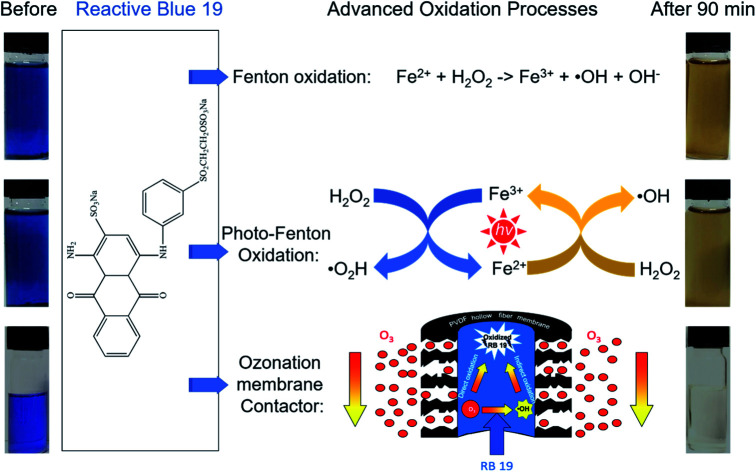

## 1. Introduction

Wastewater from textile factories is a significant source of environmental pollution. The high strength of colored effluents may cause them to become visual eyesores and causes aesthetic pollution, eutrophication and perturbations in aquatic life. In addition, color-containing dyestuffs have been found to be toxic and carcinogenic to aquatic environments.^1^ The release of colored wastewater poses a major problem for the industry as well as a threat to the environment. Therefore, it is necessary to develop an effective method for dye wastewater treatment in order to remove color from textile effluents that is both efficient and cost-effective for industries.

Advanced oxidation processes (AOPs) are an alternative approach that results in the destruction of hazardous organic compounds *via* a hydroxyl radical (˙OH), which is a highly reactive oxidant (*E*° = 2.8 V).^2^ The Fenton process is one of the most popular AOPs due to its wide applicability and simple, rapid degradation of organic compounds. The highly oxidative (˙OH) Fenton process from the reaction of hydrogen peroxide (H_2_O_2_) and the ferrous ion (Fe^2+^) under strong acidic conditions is capable of quickly degrading organic compounds that are further converted to carbon dioxide and water (complete mineralization). Fenton oxidation is considered to be an environmentally friendly treatment due to the non-toxic reagents used, its operation under ambient conditions and easy operation. Additionally, ˙OH is a non-selective oxidant, which is capable of destroying a wide range of organic pollutants in water and wastewater. Various studies were performed on the development of the Fenton oxidation processes, such as the photo-Fenton, electro-Fenton, and fluidized bed Fenton processes, for the treatment of organic compounds, and the results showed that the removal of organic compounds was achieved in 30–60 min.^3–8^ The Fenton process has been effectively used for dye wastewater treatment in recent years. Methyl orange (azo colorant) is removed from aqueous solutions by forming ˙OH from a Fe^2+^ catalyst with H_2_O_2_ after 60 min under pH 2.^9^ Sairiam *et al.* (2019) showed that Reactive Black 5 (azo dye) was completely decolorized by Fenton oxidation in 30 min.^10^ During the oxidation process, Fe^2+^ is regenerated by placing Fe^3+^ under light intensity through the photo-Fenton process, which can accelerate the decolorization of dye wastewater to maintain continuity with the previous Fenton reaction. Saleh and Taufik (2019) examined the degradation of Methylene Blue (MB) and Congo Red (CR) dyes using Fenton processes, and the results revealed that the degradation of both MB and CR dyes was activated by combining light and ultra-sonic irradiation with the Fenton process.^11^ The major drawback of the Fenton process was the sludge production (Fe^3+^) that required proper separation and a narrow operating pH range.^12^

O_3_ has been recommended in recent years as a potential alternative method for decolorization and removal of organic compounds. During the ozonation process, the ozone molecule is selective and preferentially attacks the unsaturated bonds of the chromophores, and as a result, the color is removed. The process of ozonation can take place in two ways: directly by the ozone molecule itself (O_3_) due to its high oxidation potential (*E*° = 2.07 V in an acidic medium and 1.27 V in an alkaline medium), or indirectly by changing to ˙OH. Due to the high redox potentials of the O_3_ molecule and ˙OH, the oxidation of pollutants such as organic compounds is not selective in terms of removal. It was reported that azo dyes with chromophore structures were oxidized by the ozone attack.^13^ TOC and COD were decreased in the textile wastewater by the ozonation process through oxidation by O_3_ and ˙OH.^14^ However, the low solubility of ozone in water limits the driving force, or the mass transfer of ozone gas into the aqueous phase.^15^ The dissolved O_3_ can move into the ambient environment easily because of its low Henry's constant.^16^ In addition, the low mass transfer of ozone into the aqueous phase and the low specific area limit the conventional methods of gas–liquid contact for the ozonation of wastewater, such as bubble columns and packed beds.^17^ The effectiveness of ozonation can be increased by the generation of smaller bubbles.^18^ The use of a membrane contactor is an alternative method for the ozone gas contacting process that facilitates gas movement without bubble generation by employing a hydrophobic membrane. The membrane in this process acts as a barrier between two phases, for the purpose of mass transfer between the phases, without dispersing from one phase to another. The potential advantages of a membrane contactor are its high surface area and lack of flooding.^19^ PVDF membranes are widely used as a gas distributor to produce ozone bubbles uniformly in the membrane contactor, which provides a contact surface for the oxidation of organic compounds and serves as a hydrophobic and ozone resistant membrane. Several studies have been conducted on the use of ozonation using a membrane contactor for the oxidation of organic compounds such as humic substances, phenol, acrylonitrile, nitrobenzene and dyes.^20–23^ For the membrane contacting process with ozonation, hydrophobic hollow fiber polyvinylidene fluoride (PVDF) and polytetrafluoroethylene (PTFE) membranes exhibited good Reactive Red 120, Direct Red 23 and Acid Blue 113 decolorizations, but the ozone fluxes for 16 h using a PVDF membrane were less than that for PTFE membranes due to its lower hydrophobicity.^20,21^ As a result, the PVDF membrane is wetted owing to the water penetration into the membrane pores which leads to an increase in the overall mass transfer and a decrease in membrane performance. From our previous work,^24^ a PVDF membrane modified by plasma activation followed by grafting with 1*H*,1*H*,2*H*,2*H*-perfluorodecyltriethoxysilane (PVDF-PAM) showed higher hydrophobicity and carbon dioxide gas absorption flux for 20 h in the membrane contactor system compared to the original PVDF membrane which improved the membrane wetting. Due to the high hydrophobicity of the obtained PVDF-PAM membrane,^24^ the PVDF-PAM membrane could potentially be used in the membrane contactor with ozonation for dye wastewater treatment, and this study aims to determine the efficiency and potential for its application.

Although these techniques are very effective, the determination of both appropriate AOPs and operational conditions is an important issue to resolve in order to achieve the dye decolorization at low costs. The aim of this study is to determine the applicability of Fenton oxidation and the membrane contactor with the ozonation process in the decolorization of RB 19 from synthetic dye wastewater. The effects of some operational parameters on the efficiency of the Fenton process, including initial concentrations of Fe^2+^, H_2_O_2_, RB 19 and pH on the decolorization of RB 19, were also investigated. For the membrane contactor with the ozonation process, the effect of gas velocity and liquid flow rate on the ozone flux and affecting parameters, *i.e.*, the initial pH and initial concentrations of O_3_ and RB 19, were studied. Finally, the efficiencies of RB 19 decolorization by the Fenton process and the membrane contactor with the ozonation process were compared, including decolorization efficiency and energy consumption.

## 2. Materials and methods

### 2.1 Chemicals and reagents

The dye, Reactive Blue 19 (RB 19), was kindly provided by DyStar Thai Ltd. Fenton's reagents were 30% (w/v) hydrogen peroxide (H_2_O_2_, Merck) and ferrous sulfate heptahydrate (FeSO_4_·7H_2_O, Qrec). The dye solution pH levels were adjusted using either sulfuric acid (H_2_SO_4_, Qrec) or sodium hydroxide (NaOH, Merck). Potassium titanium oxalate (C_2_K_2_O_12_Ti, Sigma Aldrich) was used to analyze the remaining H_2_O_2_ concentration. 1,10-Phenanthroline monohydrate (C_12_H_8_N_2_·H_2_O), ammonium acetate (NH_4_C_2_H_3_O_2_, Merck) and 37% hydrochloric acid (HCl, Qrec) were used to analyze the Fe^2+^ concentration.

### 2.2 Fenton oxidation procedure for RB 19 decolorization

A 500 mL sample of 75 mg L^−1^ RB 19 wastewater was prepared in 1 L for the Fenton experiment. The RB 19 wastewater was completely mixed using a magnetic stirrer (Stuart CB 162) and the pH was measured using a pH meter (UltraBasic, UB-10). In this investigation, synthetic RB 19 dye wastewater was prepared by dissolving RB 19 in deionized water (DI) and mixing with a magnetic stirrer at room temperature. The pH of the RB 19 wastewater was adjusted to the desired value by adding either H_2_SO_4_ or NaOH. Subsequently, the required amount of the ferrous (Fe^2+^) solution (as ferrous sulfate heptahydrate, FeSO_4_·7H_2_O) was added after reaching pH stability. Corresponding to this, a sample was taken at time = 0 min (before Fenton oxidation). The reaction was started by adding the H_2_O_2_ solution (30% w/v) into the RB 19 wastewater reactor. Then, the samples were taken out at time = 1, 2, 3, 4, 5, 10, 15, 20, 30, 60 and 90 min. At each time, the samples were analyzed for RB 19 concentration and the remaining concentrations of Fe^2+^ and H_2_O_2_. A 1 mL sample was taken and the reaction was stopped in 9 mL of 0.1 N NaOH to analyze RB 19. A 1 mL sample was taken and H_2_O_2_ was analyzed by the titanium oxalate method, and a 1 mL sample was taken and filtrated though a 0.45 μm membrane filter to determine the Fe^2+^ concentration *via* the 1,10-phenanthroline method. After obtaining the optimum condition from the Fenton process, the photo-Fenton process was investigated using the same method as the Fenton process. The photo-Fenton process was started by adding H_2_O_2_ and turning on the UV light (AQUA ZONIC UV mini submersible 7 Watt). In the final samples, collected after 90 min, COD and BOD_5_ were measured using the standard method. TOC and some inorganic anions were also examined using a TOC analyzer (TOC-V_CPH_, Shimadzu) and a Shodex IC NI-424 with a guard column (4.6 mm × 100 mm) and a mixture of 6 mM 4-hydroxybenzoic acid, 2.8 mM Bis–Tris, 2 mM phenylboronic acid and 0.005 mM CyDTA at a flow rate of 1 mL min^−1^ and at a temperature of 50 °C, respectively.

### 2.3 Ozonation by the membrane contacting process procedure for RB 19 decolorization

This experiment was separated into two parts. The first part aimed to study the effects of the gas velocities and liquid flow rates on ozone flux. The ozone gas was produced using an ozone generator (Model: VGsa 010, Siamese twins Ltd., Thailand) which was fed from a pure oxygen tank (Linde industrial grade, Thailand). The initial flow rate of gas containing ozone was measured using a gas flow meter (Mesa Laboratories Defender 530-M 2013, Bios International Corporation, United States). The ozone concentration was measured using an ozone analyzer (Yanco OLA/DL-5, Canada), specifically the initial and effluent concentrations of the ozone gas in the gas phase. The PVDF hollow fiber membranes used in this study were the same as those from the previous research but modified using plasma-activated membrane modification (PVDF-PAM) and the membrane properties used in this study were reported in Sairiam *et al.* (2013).^24^ The membrane module was prepared by placing 30 fibers of PVDF hollow fiber membranes in a glass tube with an inner diameter of 10 mm and effective length of 25 cm as reported in Table 1.

Specifications of hollow fiber membranes and the module

**Table:** 

Membranes	
Fiber o.d. (mm)	1.16
Fiber i.d. (mm)	0.8
Membrane pore size (μm)	0.14
Membrane porosity (%)	80
Number of fibers	30
Effective contact area (m^2^)	0.01885
Contact angle (°)	145

**Table:** 

Module	
Module o.d. (mm)	12
Module i.d. (mm)	10
Effective module length (mm)	250

**Fig. 1:**
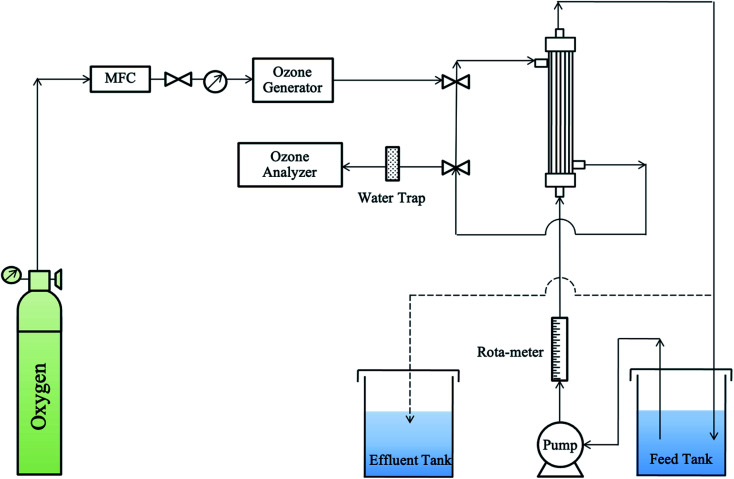
The schematic diagram of a gas–liquid membrane contactor system.

Then, the initial ozone gas was transferred to the shell side of the membrane module and dispersed into the liquid phase during the ozonation process. The liquid phase of this study was the synthetic RB 19 wastewater. The RB 19 wastewater was fed into the lumen side of the PVDF hollow fiber membrane module. Moreover, the liquid flow rates were controlled using a peristaltic pump (Masterflex L/S® Easy-Load® II, United States). The first part was presented as a single pass as exhibited by the normal line in Fig. 1. The study used gas velocities that varied from 0.13 to 0.53 m s^−1^. The liquid gas flow rates were studied from 200 to 500 mL min^−1^. Both of the parameters were determined in 5 min of the ozonation process because of the higher stability of ozone gas in the ozone flux. Thus, the ozone fluxes were calculated by the mass balance of ozone utilization in the gas phase at different gas and liquid velocities using eqn (1) and (2):1
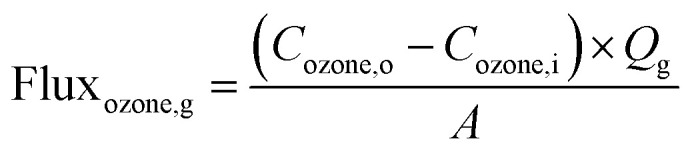
2
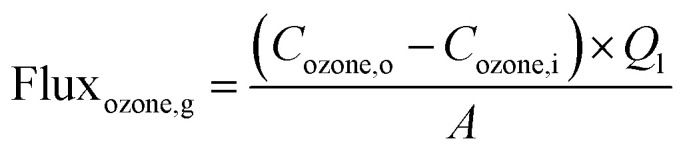
where flux_ozone,g_ is the flux of ozone in the gas phase, *C*_ozone,o_ is the ozone concentration (mg L^−1^), *C*_ozone,i_ is the outlet ozone concentration (mg L^−1^), *Q*_g_ is the gas flow rate, *Q*_l_ is the liquid flow rate (mL min^−1^) and *A* is the effective surface area of hollow fiber membranes in the module.

The second part aimed to study the decolorization efficiency and was similar to the first part. The effluent of RB 19 wastewater was recycled through the membrane module, and then fed back to the feed tank for 90 minutes of the ozonation process as shown by the dashed line in Fig. 1.

All of the experimental procedures for the second part were performed in a 2 L capacity glass beaker (Pyrex®, Germany) under room conditions. The decolorization efficiencies in this part were determined according to the results of the first part (gas velocity and liquid flow rate). The pH values were varied from pH 3 to 11 and measured using a pH meter (Horiba pH meter D-12, Japan). The ozone concentrations investigated ranged from 30 to 50 mg L^−1^. The RB 19 dye concentrations were studied from 0.04 to 0.16 mM. In addition, samples were taken at 0, 1, 2, 3, 4, 5, 10, 20, 30, 60, and 90 min. After a treatment time of 90 min, the samples were collected to analyze as mentioned in Section 2.2.

### 2.4 Analytical methods

#### 2.4.1 Hydrogen peroxide (H_2_O_2_) concentration

The H_2_O_2_ concentration was determined using potassium titanium oxalate in acidic solution as a reactant which was based on the generation of a yellow–orange titanium(iv) peroxide complex and using a spectrophotometer (Genesys) at a wavelength of 400 nm.^25^ A calibration curve was plotted using standard H_2_O_2_ with known concentrations to determine the hydrogen peroxide concentration that remained.

#### 2.4.2 Ferrous (Fe^2+^) concentration

The concentration of ferrous ions (Fe^2+^) was determined using 1,10-phenanthroline monohydrate by measuring the light absorbance at a wavelength of 510 nm using a spectrophotometer (Genesys). A calibration curve was plotted using a standard ferrous solution with known concentrations to determine the Fe^2+^ concentration.

#### 2.4.3 Dye concentration

For the Fenton experiment, a dye sample was taken out and then mixed with 0.1 N NaOH to stop the reaction. Dye concentrations were analyzed using a spectrophotometer (Genesys) with a maximum absorbance wavelength (*λ*_max_) of RB 19 at 595 nm with a plastic spectrometric quartz cell (1 cm path length). The standard curve of RR 239 was constructed to determine the concentration. The decolorization efficiency (%) of RB 19 was defined by the following equation.3
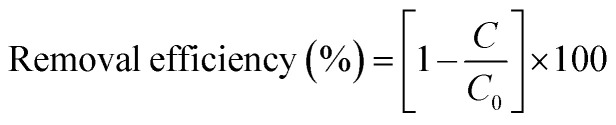
where *C* (mg L^−1^) is the concentration of color at reaction time and *C*_0_ (mg L^−1^) is the initial concentration of color.

## 3. Results and discussion

### 3.1 Influence of Fenton reagents on RB 19 decolorization

The different initial concentrations of Fe^2+^, H_2_O_2_, RB 19 and pH tested are summarized in Table 2. It can be observed that a positive influence of affecting parameters on RB 19 decolorization was obtained.

**Table:** RB 19 decolorization achieved for the different concentrations of Fenton's reagent, pH and initial RB 19 concentration

Run no.	pH	[Fe^2+^] (mg L^−1^)	[H_2_O_2_] (mM)	[RB 19] (mg L^−1^)	RB 19 decolorization (%)
1	2	0.25	1.5	75	91.0
2	3	0.25	1.5	75	91.2
3	4	0.25	1.5	75	85.1
4	5	0.25	1.5	75	85.5
5	3	0.05	1.5	75	76.6
6	3	0.15	1.5	75	88.9
7	3	0.35	1.5	75	84.6
8	3	0.25	0.5	75	64.6
9	3	0.25	2.5	75	93.8
10	3	0.25	3.5	75	97.0
11	3	0.25	1.5	25	96.3
12	3	0.25	1.5	50	91.3
13	3	0.25	1.5	100	85.3

#### 3.1.1 Effect of initial pH

**Fig. 2:**
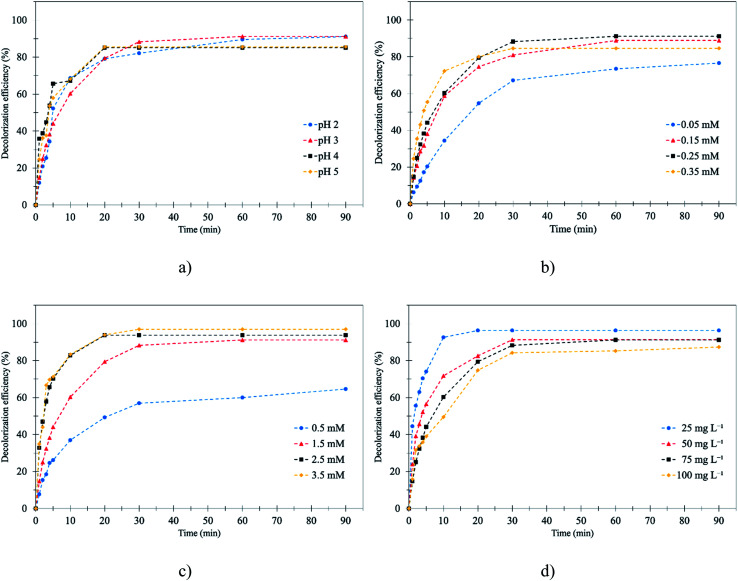
Effect of affecting parameters on the Fenton process for RB 19 decolorization: (a) pH; (b) initial Fe^2+^ concentration; (c) initial H_2_O_2_ concentration; and (d) initial RB 19 concentration.

The pH is an important factor for the Fenton process.^26^ Therefore, the initial pH that would achieve the highest decolorization efficiency was determined. The initial pH was studied by varying the level from 2 to 5 at 0.25 mM Fe^2+^ and 1.5 mM H_2_O_2_ at room temperature for 90 min. According to the results, pH 3 was found to be the optimum pH for decolorization of RB 19 by the Fenton process. It was noted that, at 0–10 min, the RB 19 was decolorized very quickly (60–70%) because of the Fenton reaction as illustrated in Fig. 2a. This was because Fe^2+^ reacts with H_2_O_2_ very quickly to generate the hydroxyl radical (˙OH).^27^ Then, RB 19 molecules can be oxidized by ˙OH as shown in eqn (4)–(6) as follows:^28^

4 Fe^2+^ + H_2_O_2_ → Fe^3+^ + ˙OH + OH^−^ (*k*_1_ = 76 M^−1^ s^−1^)

5 Organic + ˙OH → ˙Organic

6 ˙Organic → CO_2_ + H_2_O

The results show that the decolorization efficiency slightly increased from 91.0% to 93.8% by increasing the pH from 2 to 3. According to the results, at pH 2, the reaction had a lower decolorization efficiency than at pH 3 because a proton (H^+^) in solution reacted with hydrogen peroxide (H_2_O_2_) and an oxonium ion (H_3_O_2_^+^) was formed and reduced the electrophilicity of H_2_O_2_.^29^ Moreover, at a lower pH, the decolorization efficiency decreased because ˙OH was scavenged by hydronium ions^8^ as shown in eqn (7) and (8).

7 H_2_O_2_ + H^+^ → H_3_O_2_^+^

8 ˙OH + H^+^ → H_2_O

Nevertheless, the RB 19 decolorization efficiency decreased from 93.8% to 85.5% when the pH was increased from 3 to 5. This was because the Fe^2+^, which was the substrate for the Fenton process, formed into Fe(OH)_2_^+^, Fe(OH)_3_, and Fe(OH)_4_^−^.^30^ Moreover, at a higher pH the generated hydroxide could decompose and finally become oxygen.^31^ This result was similar to the previous study on the decolorization of Reactive Red 239 by Fenton oxidation that the RR 239 decolorization efficiency decreased when the pH increased from 3 to 6.25.^32^ Thus, the optimal conditions for efficient RB 19 removal were obtained at pH 3.0 with 93.8% decolorization.

#### 3.1.2 Effect of initial ferrous concentration (Fe^2+^)

Ferrous concentration (Fe^2+^) plays the main role in the formation of hydroxyl radicals (˙OH) which affects the efficiency of Fenton oxidation.^33^ In this study, initial Fe^2+^ dosage was varied from 0.05 to 0.35 mM with fixed RB 19 and H_2_O_2_ concentrations of 0.12 mM and 1.5 mM, respectively, and at pH 3.0. The decolorization efficiency increased from 76.6% to 91.2% with an increase in ferrous concentration from 0.05 mM to 0.25 mM as illustrated in Fig. 2b. This was because at low Fe^2+^ concentration, the production of ˙OH was inadequate and Fe^2+^ could not completely react with H_2_O_2_ to form ˙OH. Therefore, at low Fe^2+^ concentration, the RB 19 decolorization was low. A higher Fe^2+^ dosage generated more ˙OH through the mixture of Fe^2+^ with H_2_O_2_.^34^ In this study, the highest RB 19 decolorization obtained corresponded to the H_2_O_2_/Fe^2+^ ratio = 14, but the theoretical molar ratio should be 11. However, the optimal molar ratio was different for the decolorization of different dyes. As a result, RB 19 was oxidized by ˙OH and color molecules were removed as presented in eqn (4)–(6). Although the Fe^2+^ concentration increased from 0.25 mM to 0.35 mM, the decolorization efficiency decreased obviously from 91.2% to 84.6%. This was because of the excess Fe^2+^ that might have caused a scavenging effect on the ˙OH. This implies that increasing the Fe^2+^ concentration of H_2_O_2_/Fe^2+^ above 6 did not enhance the decolorization efficiency of RB 19, probably due to the ˙OH scavenging effect in the presence of excess Fe^2+^. The Fenton reaction produced significant ˙OH which caused scavenging effects and generated H_2_O_2_ as shown in eqn (9) and (10).

9 Fe^2+^ + ˙OH → Fe^3+^ + OH^−^

10 ˙OH + ˙OH → H_2_O_2_

The results also showed that the color removal increased rapidly in the first 10 minutes. This process can be described in two different steps. In the first step, the ˙OH was formed by the reaction between H_2_O_2_ and Fe^2+^ (*k*_1_ = 76 M^−1^ s^−1^). The Fe^2+^ in the RB 19 wastewater decreased rapidly in first 10 minutes as presented in Fig. 2b. Then, the RB 19 decomposed very quickly by the ˙OH; in contrast, ferric ions (Fe^3+^) were produced in this step.^35^ After 30 min, the decolorization rate decreased, because the second stage had begun due to the reaction of Fe^3+^ and H_2_O_2_ (*k*_2_ = 0.01–0.02 M^−1^ s^−1^) in eqn (11). Hydroperoxyl radicals (˙O_2_H) were produced and could degrade the RB 19, but the reaction rate in this stage was slower than in the first stage. Therefore, the decolorization rate decreased.^36^ This result was similar to the decolorization of Acid Yellow 23 by Fenton and photo-Fenton oxidation, in which the concentration of Fe^2+^ increased from 0.002 mmol to 0.12 mmol, and as a result, the decolorization of Acid Yellow 23 increased from 30% to nearly 100% due to the generation of ˙OH from Fe^2+^.^37^

11 Fe^3+^ + H_2_O_2_ → Fe^2+^ + H^+^ + ˙O_2_H (*k*_2_ = 0.01–0.02 M^−1^ s^−1^)

#### 3.1.3 Effect of initial H_2_O_2_ concentration

H_2_O_2_ dosage also plays an important role in the efficiency of the Fenton process as a consequence of the generation of ˙OH.^38^ Therefore, the effect of initial dosage was investigated by testing the effect of its variation from 0.5 mM to 3.5 mM on the efficiencies of the Fenton process in decolorization of RB 19 dye wastewater, with fixed 0.12 mM RB 19, 0.25 mM Fe^2+^ and pH 3 for 90 min as illustrated in Fig. 2c. The results showed that the color removal efficiency increased from 64.6% to 91.2% with an increase in H_2_O_2_ dosage from 0.5 mM to 1.5 mM. The decolorization slightly increased from 91.2% to 97.0% after an increase in H_2_O_2_ from 1.5 mM to 2.5 mM and 3.5 mM. It could be seen that the decolorization efficiency of RB 19 increased distinctly with an increasing concentration of H_2_O_2_. Therefore, addition of H_2_O_2_ at a concentration higher than 1.5 mM did not significantly affect the decolorization efficiency. These findings were similar to the results of the decolorization of Reactive Blue 4 (RB 4) by Fenton oxidation, in which an initial H_2_O_2_ concentration of 8 mM was reported as the optimum concentration, and the decolorization of RB 4 increased from 80% to 96% with an increase in H_2_O_2_ dosage from 4 mM to 8 mM.^39^ This seems to indicate that excessive H_2_O_2_ has a scavenging effect on ˙OH as shown in eqn (7), which produced the hydroperoxyl radical (˙O_2_H). The ˙O_2_H species had much weaker oxidizing power compared with ˙OH. Also, an excess amount of H_2_O_2_ could decompose to oxygen and water. Therefore, the excess ˙OH in the solution may have recombined and become H_2_O_2_.^40^ According to the results, it was concluded that the optimal H_2_O_2_/Fe^2+^ ratio for RB 19 decolorization was 14, which was higher than the theoretical ratio. However, the optimal molar ratio is different in various dyes, *e.g.* 67 : 1 for Reactive Red 239,^32^ 50 : 1 for Reactive Black 5 ^10^ and 11 : 1 for Drimaren Orange HF 2GL.^41^ The optimal ratio was therefore obtained when neither Fe^2+^ nor H_2_O_2_ was overdosed.

#### 3.1.4 Effect of initial RB 19 concentration

The RB 19 concentration of the wastewater was related to Fenton's reagent because the pollutant molecule is also an important parameter for wastewater treatment.^42^ The effects of initial dye concentrations (25, 50, 75 and 100 mg L^−1^) on the decolorization efficiencies of RB 19 were studied at fixed 1.5 mM H_2_O_2_, 0.25 mM Fe^2+^ and at pH 3 for 90 min. The results showed that the decolorization efficiency decreased from 96.3% to 85.3% as the dye concentration increased from 25 mg L^−1^ to 100 mg L^−1^ as illustrated in Fig. 2d. Similar results were reported, demonstrating that phenol removal by the Fenton and electro-Fenton processes decreased from 98.9% to 68.6% when initial phenol concentrations increased from 50 to 500 mg L^−1^, owing to the increase of the dye concentration but the same concentration of ˙OH.^43^

### 3.2 Influence of ozonation parameters operating with the membrane on RB 19 decolorization

#### 3.2.1 Effect of gas velocity

The effect of gas velocity on the ozone flux was determined by its variation from 0.13 to 0.53 m s^−1^ for 0.075 mM RB 19 as presented in Fig. S1.† The results found that as the gas velocity increased, the ozone flux increased from 1.33 to 3.72 mg m^−2^ s^−1^. In addition, it was reported that an increase in gas velocity to over 50 L h^−1^ or 0.74 m s^−1^ had an insignificant effect on the decolorization efficiency, since the increased gas velocity caused the ozone utilization to decrease.^44^ The ozone utilization decreased when the gas velocities were over 0.27 m s^−1^. Bamperng *et al.* (2010) studied the ozone fluxes at varied gas velocity (0.12–0.22 m s^−1^) with 40 mg L^−1^ ozone concentration and the results revealed that ozone flux was not influenced.^21^

#### 3.2.2 Effect of liquid velocity

The study examined the effect of liquid flow rates ranging from 200 to 500 m L^−1^ on ozone flux. The results showed that the ozone flux increased from 1.95 to 5.76 mg m^−2^ s^−1^ as the liquid flow rate increased from 200 to 500 mL min^−1^ because of its solubility in water as illustrated in Fig. S1.† The ozone utilization was constant at liquid flow rates over 400 mL min^−1^, because the decolorization efficiency decreased at higher liquid flow rates due to the shorter time for the ozonation process.^44^ As seen previously, the liquid phase mass transfer coefficient increased with the increase in liquid flow rate.^20,21,45^ Thus, the appropriate liquid flow rate was 400 mL min^−1^ for the decolorization of RB 19.

#### 3.2.3 Effect of ozone concentration

**Fig. 3:**
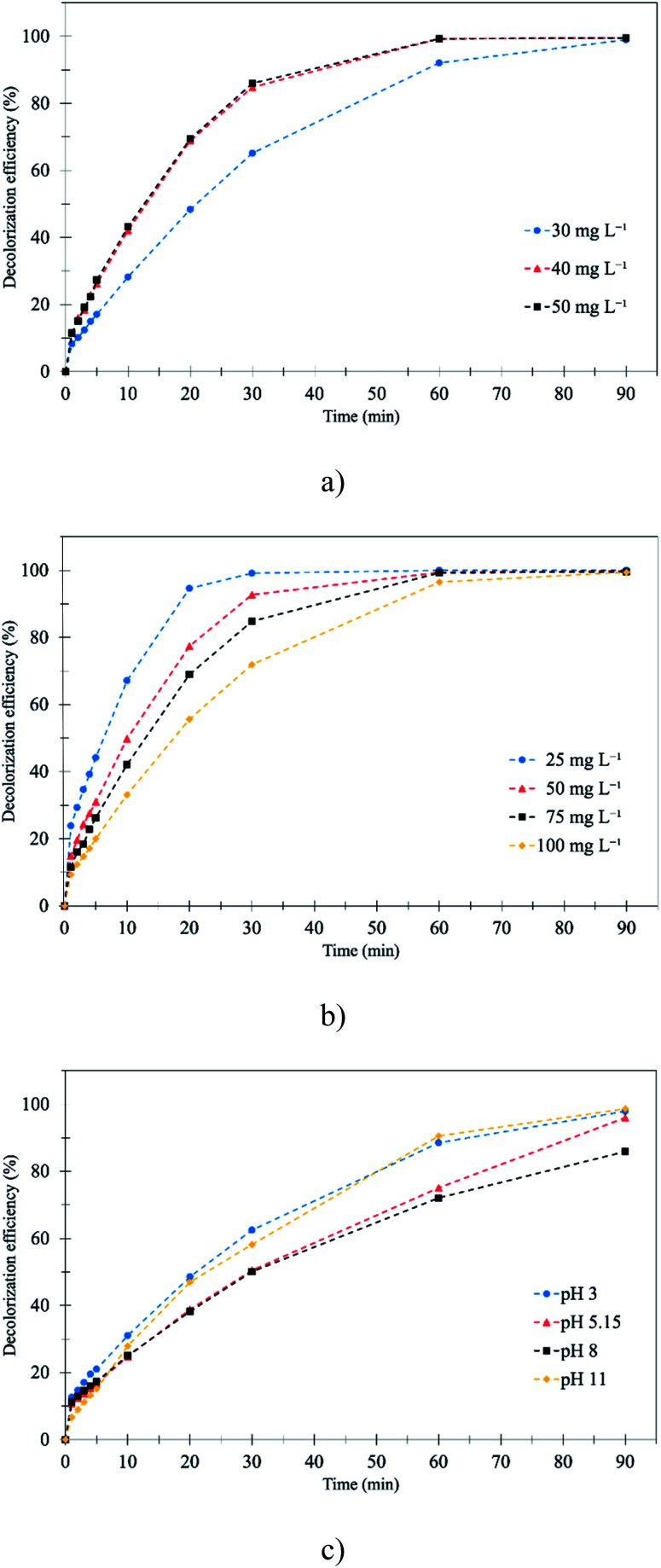
Effect of affecting parameters on RB 19 decolorization by the membrane contacting process with ozonation: (a) initial ozone concentration; (b) initial RB 19 concentration; and (c) initial pH.

The obtained results showed that the gas velocity and liquid flow rate affected the ozone flux. Thus, the 400 mL min^−1^ liquid flow rate and 0.27 m s^−1^ gas velocity from the previous part were chosen for studying the effect of ozone concentrations, initial RB 19 concentrations and pH. The decolorization of RB 19 using the membrane contacting process with ozonation at varied ozone concentrations is summarized in Table 3. Ozone concentrations ranging from 30 to 50 mg L^−1^ with different initial RB 19 concentrations were studied. The results showed that an increase in ozone concentration increased the decolorization efficiency. For instance, the RB 19 (0.12 mM) was decolorized to 99.0, 99.6 and 99.6% when ozone concentrations of 30, 40 and 50 mg L^−1^, respectively, were applied for 90 min as presented in Fig. 3a. For 10 min of the ozonation process, it was noticed that the decolorization was very fast. The decolorizations of RB 19 were 28.2, 42.1 and 43.2% with ozone concentrations of 30, 40 and 50 mg L^−1^, respectively. This was because the predominance of the direct ozonation process by the ozone molecule itself led to the destruction of RB 19 by the increase in ozone concentration.^46^ The reactive O_3_ molecule was sufficiently promoted to oxidize the RB 19 at the selective site. Stylianou, Kostoglou, and Zouboulis (2016) showed that the dissolved ozone concentration in the liquid phase increased with the concentration of ozone gas, resulting in the increase of partial pressure by Henry's law at the interface between the gas and liquid.^47^ Moreover, the performances of decolorization efficiency at ozone concentrations of 40 and 50 mg L^−1^ for the entire ozonation process were similar. Excess O_3_ concentration seemed to inhibit the direct oxidation of RB 19, which presented unchanged RB 19 decolorization. Therefore, 40 mg L^−1^ ozone concentration was selected to study further.

**Table:** RB 19 decolorization achieved for the different initial concentrations of O_3_ and RB 19, and different pH

Run no.	pH	[O_3_] (mg L^−1^)	[RB 19] (mg L^−1^)	RB 19 decolorization (%)
1	5.15	30	75	99.0
2	5.15	40	75	99.6
3	5.15	50	75	99.6
4	5.15	40	25	100.0
5	5.15	40	50	99.8
6	5.15	40	100	99.5
7	3	40	75	97.9
8	8	40	75	85.9
9	11	40	75	98.6

#### 3.2.4 Effect of initial RB 19 concentration

The different initial RB 19 concentrations (0.04–0.16 mM) were studied at a fixed ozone concentration of 40 mg L^−1^. The results showed that the wastewater containing an initial RB 19 concentration of 0.04 mM was completely decolorized after 60 minutes. The decolorization decreased from 100% to 96.5% when the initial RB 19 concentration was increased from 0.04 to 0.16 mM as illustrated in Fig. 3b and Table 3. This was because the number of molecules increased with the increased RB 19 concentration, but the ozone molecules stayed constant, resulting in insufficient ozone molecules for complete decolorization.

#### 3.2.5 Effect of initial pH

The decolorization efficiencies at pH 3, 5.15, 8 and 11 were 97.9, 96.0, 85.9 and 98.6%, respectively, at 90 minutes as presented in Table 3. The results showed that the decolorization efficiencies of pH 3 and 11 were the highest after the ozonation process as depicted in Fig. 3c. According to the results, the direct ozonation process was predominated by the ozone molecule at pH 3 and 5.15, while ozone molecules were decomposed to ˙OH under alkaline conditions at pH 8 and 11. At pH 8, neither O_3_ nor ˙OH was the dominant oxidizing agent, and the oxidation of RB 19 was not highly efficient as compared to the extreme conditions at pH 3 (O_3_) and pH 11 (˙OH). Then, the dissolved O_3_ gradually decreased with an increase in the initial pH of RB 19, resulting in the reduction of the reaction between ˙OH and RB 19. The highest decolorization efficiencies were obtained at pH 3 and 11. Moreover, the results at an early stage (30 min) of the ozonation process as presented in Fig. 3c showed that at pH 3 the decolorization of RB 19 was slightly more effective than at pH 11. This was because the produced HO_2_˙ acted as a scavenging reagent which reacted with the ˙OH as in eqn (9), and the remaining structure of anthraquinone dye was insensitive to the oxidation reaction, a leuco form in an acidic medium with high solubility.^48^ In addition, the RB 19 structure during the 10 min ozonation process, which was characterized by FTIR spectra, showed the cleavages of anthraquinone rings, the connecting bonds and the vinylsulfonyl group (–SO_2_CH_2_CH_2_OSO_3_Na).^46^ The generation of intermediate compounds after 30 min of the ozonation process was found by the insignificant increase of decolorization efficiencies under the alkaline conditions (pH 11) as shown in eqn (12).

12 O_3_ + OH^−^ → O_2_^−^˙ + HO_2_˙

### 3.3 Comparison of Fenton, photo-Fenton and ozonation by a membrane contactor for RB 19 decolorization

**Fig. 4:**
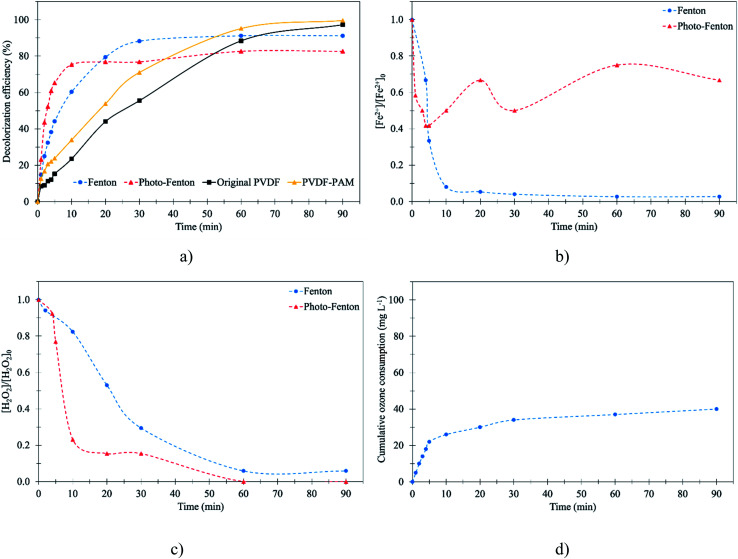
Decolorization efficiency of RB 19 under [RB 19] = 75 mg L; [H_2_O_2_] = 1.5 mM; [Fe^2+^] = 0.25 mM; pH = 3.0; [O_3_] = 40 mg L^−1^: (a) decolorization performance by different methods; (b) the remaining Fe^2+^ concentration; (c) the remaining H_2_O_2_ concentration; and (d) cumulative ozone consumption.

According to the results from the previous part, the decolorization efficiency obtained by the Fenton process depended on the Fenton's reagents. It was assumed that the combination of Fenton oxidation and UV radiation, called photo-Fenton oxidation, generates high ˙OH and regenerated Fe^2+^ compared to ordinary Fenton oxidation, leading to the facilitation of the oxidation of the RB 19 molecule. For this reason, photo-Fenton oxidation was executed under the same conditions as ordinary Fenton oxidation for comparison. Fenton and photo-Fenton oxidation were compared at pH 3. The photo-Fenton experiments were performed with 7 watts of UV light for 90 min, and the results showed that in the first 10 min the decolorization of RB 19 was 75.4%. In contrast, the decolorization of RB 19 was only 60.3% by the Fenton process as depicted in Fig. 4a. It was also found that the decolorization of RB 19 by the photo-Fenton process was slightly higher in comparison with the Fenton process. This indicated that the decolorization of RB 19 by breaking down RB 19 with ˙OH could be strongly facilitated by ultraviolet light (UV) irradiation due to the high generation of ˙OH by photo-Fenton oxidation. In this case, the photolysis of Fe^3+^ complexes, *i.e.*, Fe(OH)_2_^+^, Fe(OH)_3_, and Fe(OH)_4_^−^, allows Fe^2+^ regeneration as described in eqn (13)–(17).^49^ In addition, the formation of ˙OH also took place by breaking the H_2_O_2_ molecule *via* photolysis in eqn (14),^50^ and as shown in the results, the remaining H_2_O_2_ after photo-Fenton oxidation was lower than that in Fenton oxidation as depicted in Fig. 4c.

13 Fe^3+^ + H_2_O + *hν* → Fe^2+^ + ˙OH + H^+^

14 Fe^3+^ + H_2_O_2_ + *hν* → Fe^2+^ + ˙HO_2_ + H^+^

15 Fe^3+^ + ˙HO_2_ → Fe^2+^ + O_2_ + H^+^

16 Fe^3+^ + H˙ → Fe^2+^ + H^+^

17 H_2_O_2_ + *hν* → 2˙OH

At 20 min, the decolorization of RB 19 by Fenton oxidation was higher than by photo-Fenton oxidation. At a higher concentration of Fe^2+^ due to the Fe^2+^ regeneration *via* photo assistance, as shown in Fig. 4b, the extent of RB 19 decolorization might become constant owing to the consumption of ˙OH by Fe^2+^ as described in the previous paragraph. Finally, RB 19 could be degraded by 91.2% by Fenton oxidation and 82.6% by photo-Fenton oxidation as presented in Fig. 4a. The excessive Fe^2+^ was attributed to the scavenging effect as described in eqn (13)–(17), as the faster regeneration of Fe^2+^ reported in Fig. 4b led to the higher Fe^2+^ concentration. As a result, more Fe^2+^ was available for the ˙OH reaction by Fe^2+^, and then the decolorization of RB 19 was the negative effect. This result was similar to the previous study's findings that photo-assisted photo-Fenton did not enhance the decolorization efficiency and rate kinetic constant due to the overdose of Fe^2+^ regeneration with the increase of light intensity during the photo-Fenton oxidation of RR 239.^32^

For the membrane contacting process under the optimal conditions obtained in Section 3.2, at pH 3, the results revealed that the decolorization efficiency gradually increased with time. The decolorization efficiency of RB 19 by ozonation with the membrane contactor for 60 min was lower than that by the Fenton and photo-Fenton processes. Due to the lower oxidation potential of O_3_ (2.07 eV at pH 3), a lower decolorization of RB 19 was observed during the first 60 min. By contrast, in both the Fenton and photo-Fenton oxidations, the generated ˙OH had the highest oxidation potential and oxidized the RB 19 to a larger extent as compared to RB 19 decolorization by the O_3_ oxidizing agent in the ozonation membrane contactor. However, after 90 min operating time, the decolorization efficiency of RB 19 by the membrane contacting process with the ozonation process (98.6%) was higher than that by the Fenton process (91.2%) as presented in Fig. 4a. This result confirmed that the oxidation of RB 19 by ozone at pH 3 in the aqueous solution could decolorize the RB 19. This was because the ozone molecule dominates the oxidation process under low pH and is selective, preferentially attacking the unsaturated bonds of chromophores. It was also observed that decolorization of RB 19 was almost complete at 100% after 90 min ozonation time, but 48.5% of the colors were removed in the first 20 min as explained above. This result was similar to Acid Blue 80 (AB 80) decolorization *via* Fenton oxidation and ozonation processes; the results found that AB 80 decolorization by Fenton oxidation was rapid at the beginning for 1 min, but after 3 min the ozonation process was more efficient.^51^ It was observed that in ozonation with the membrane contactor, the membrane played an important role in RB 19 decolorization. The PVDF-PAM membrane performed better than the original PVDF membrane. This was because the PVDF-PAM membrane was more hydrophobic than the original PVDF membrane.^24^ As a result, O_3_ molecules could promote the RB 19 reduction with the PVDF-PAM membrane due to the unwetted pores of the membrane leading to an increase in the ozone mass transfer to oxidize RB 19 to small molecules.

The use of ozone and the removal of RB 19 were rapid as the ozonation treatment operated as shown in Fig. 4d. The result showed that the decolorization of RB 19 was constant by Fenton oxidation, and in contrast, the decolorization of RB 19 increased *via* ozonation with the membrane contacting process due to the ozone generation and consumption. Ozone consumption is defined as the utilization of ozone molecules for RB 19 oxidation and invalid decomposition. The cumulative ozone was affected for RB 19 decolorization. RB 19 decolorization was investigated for time of decolorization as well as ozone used. As seen in Fig. 4d, at pH 3 and an initial ozone concentration of 40 mg L^−1^, the cumulative ozone consumption increased markedly in the first 10 min. The cumulative ozone consumption after 90 min was 40 mg L^−1^. The colors of RB 19 after 90 min of treatment are presented in Fig. S2.† The results revealed that the color of RB 19 after 90 min using the membrane contactor was clear, but the colors of RB 19 by the Fenton oxidations were still yellowish. Therefore, the reaction between the RB 19 molecule and the ozone proceeded slowly and required a significant time for complete degradation.

### 3.4 Mineralization and formation of inorganic ion species

**Fig. 5:**
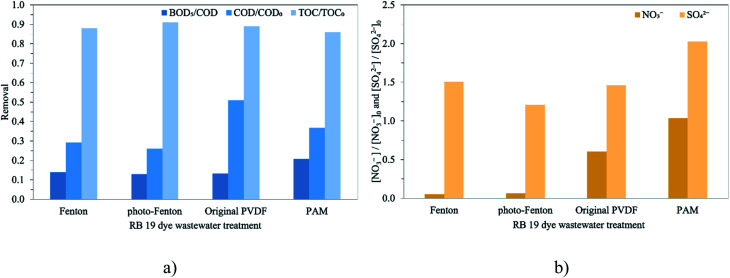
Degradation of RB 19 by Fenton oxidations and ozonation with the membrane contactor: (a) COD, BOD5 and TOC removals, and (b) inorganic anion removal.

The general reaction mechanism for RB 19 mineralization by oxidative reaction occurred either by ˙OH *via* Fenton oxidation or indirect ozonation oxidation or by O_3_ molecules by direct ozonation oxidation. The observed decreases in COD and TOC account for the mineralization process, in which a molecule is oxidized to mineral anion ions such as NO_3_^−^ and SO_4_^2−^ as described in Fig. 5. COD and TOC reductions *via* Fenton oxidations occur quickly in the first 5 min of the reaction, and then the removal rate slows and the removals are approximately constant until 90 min. In contrast, for the ozonation process with the membrane contactor using either the original PVDF or PVDF-PAM membranes, the COD removal was rapid during the first 4 min and slightly increased up to 90 min as depicted in Fig. S3.† The COD/COD_0_ ratios for RB 19 degradation *via* Fenton oxidation (approximately 70%) showed higher efficiency than ozonation with the membrane contactor (50–60%), as illustrated in Fig. 5a. The COD/COD_0_ ratio for RB 19 degradation through Fenton oxidation was not enhanced by photo assistance, which was related to the RB 19 decolorization. This was possibly due to the regeneration of Fe^2+^ in the system that would act as scavenging ions to react with ˙OH. The Fe^2+^ regeneration by photo-assisted Fenton oxidation was more than seven times greater than by ordinary Fenton oxidation, as shown in Fig. 4b. It was more likely that Fenton oxidations occur *via* the form of ˙OH, which has higher oxidation potential than O_3_. It was observed that in ozonation with the membrane contactor, the membrane played an important role in reducing COD. The PVDF-PAM membrane performed better than the original PVDF membrane. As a result of the highly hydrophobic PVDF-PAM membrane, the COD reduction was higher than that using the original PVDF membrane, leading to oxidization of RB 19 to small molecules. In oxidation with ˙OH and O_3_, almost complete decolorization required the cleavage of all chromophores in RB 19, which hardly contributes to COD removal. The decrease in COD indicated that the progress of oxidation was explained by the conversion of aromatic compounds into aliphatic compounds by ring-opening reactions.

The TOC profiles for the experiments carried out for the different treatments of RB 19 are shown in Fig. S3.† Despite the high removal efficiencies of RB 19 and COD obtained, the process was not efficient for removing TOC. TOC reductions were fast in the first 10 min, and then the removal rates remained constant until 90 min. A similar trend was observed for RB 19 and COD removals. The maximized removal was only 14%, indicating that mineralization was not complete as shown in Fig. 5a. Comparing the TOC removal, there was no significant difference among treatments. The differences in TOC removal among treatments are small and do not correlate with ozone and ˙OH cleavage ability. In addition, the BOD_5_/COD ratio slightly increased to 0.13–0.21 compared to the 0.10 in the untreated RB 19 wastewater. This finding showed that the highest BOD_5_/COD ratio was achieved with the ozonation membrane contactor using the PVDF-PAM membrane, meaning some increasing biodegradability. It implied that the degradation of RB 19 by the ozone molecule itself through the ozonation process produced non-toxic compounds compared to Fenton oxidation by ˙OH. The BOD_5_/COD ratio is more relevant than the final BOD_5_ since it expresses the biodegradable organic fraction of the total organic compounds. This indicated that despite the unsuccessful mineralization of RB 19, it could break down the components into biodegradable compounds.

**Fig. 6:**
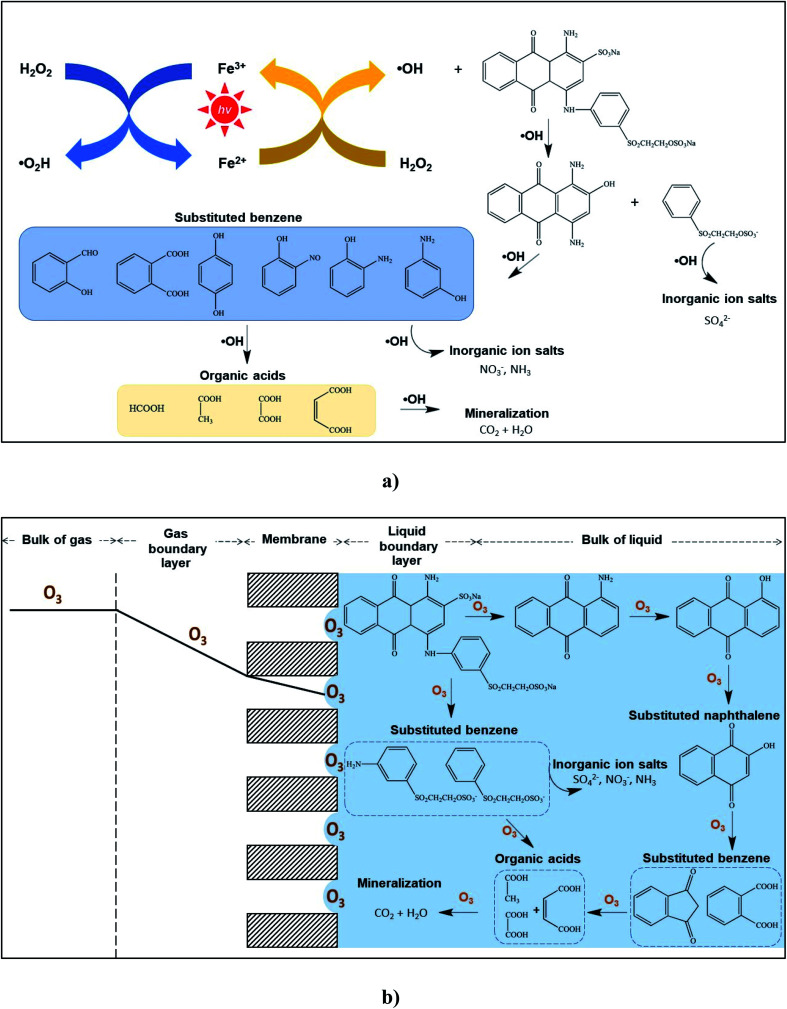
Possible mechanism and degradation pathways of RB 19 under acidic conditions:^46,52–54^ (a) Fenton oxidations and (b) ozonation oxidation in the membrane contactor.

Nitrogen (N^−^) and sulfur (S^−^) present in the RB 19 structure could be formed during the treatments due to the main inorganic molecule as the mineralized ionic compounds. The evaluations of NO_3_^−^ and SO_4_^2−^ were performed for RB 19 degradation by the Fenton oxidations and ozonation process with the membrane contactor as depicted in Fig. 5b, resulting from the oxidation and cleavage of the sulfonic and nitrogen groups presented in RB 19. The possible pathways of intermediates formed during the cleavage of RB 19 by Fenton oxidations and ozonation process are illustrated in Fig. 6a and b. Degradation of RB 19 occurs through the ˙OH mechanism and direct O_3_ oxidation following the Fenton oxidation and ozonation process, respectively. As the decolorization is very fast, the attack of O_3_ under acidic conditions took place for RB 19 destruction to 1-amino anthraquinone.^46,52^ In contrast, 1,4-diamino-2-hydroxyanthra-9,10-quinone was formed *via* the reaction of ˙OH *via* Fenton oxidation.^53^ Hence, the fragments produced by the cleavage of RB 19 might be the primary reaction intermediates including substituted naphthalene and substituted benzene. Phthalic acid and other substituted aromatic compounds have been reported during the ozonation process of RB 19. Then, phthalic acid was further oxidized to smaller organic acids such as butene diacid, oxalic acid, and acetic acid and mineralized to CO_2_ and water.^46,52–54^

Additionally, the concentrations of NO_3_^−^ and SO_4_^2−^ increased after treatment, and the RB 19 oxidized by the O_3_ molecule gave higher NO_3_^−^ and SO_4_^2−^ than the ˙OH by the Fenton oxidations, illustrated in Fig. 5b. This observation strongly indicated that the existing NO_3_^−^ might be converted from oxidation. From the substituted benzene ring, NO_2_^−^ was directly or oxidized initially and then formed NO_3_^−^. This likely means that the nitrogen linked to the benzene ring of RB 19 is more reactive, leading to the rapid formation of NO_3_^−^ as depicted in Fig. 6. Ozone initially attacked the amino groups because of its electrophilic characteristics, which means that it readily reacts with the negatively charged nitrogen atom followed by oxidization to form nitro groups.^46^ It was also observed that the cleavage of the sulfonic group of RB 19 leads to an increase in SO_4_^2−^, implying that the vinyl sulfonyl group (–SO_2_CH_2_CH_2_OSO_3_Na) on the mono benzene ring, or sulfonic group (–SO_2_Na) on the anthraquinone rings of RB 19, was partially oxidized during the treatments through either ˙OH or O_3_. This result was similar to the nitrogen linked to the naphthalene ring of RB 5 which was oxidized to ammonia and finally changed to nitrate and nitrite ions by ˙OH.^55,56^

Table 4 summarizes the related methods for RB 19 decolorization which were Fenton and ozonation oxidations with their conditions. The decolorizations of RB 19 by Fenton oxidation and ozonation processes were well fitted with the pseudo-first-order model.^53,54^ Under the initial pH of RB 19 solution without pH adjustment for decolorization by the ozonation process, the TOC removal efficiency was 90% at 55.8 mg L^−1^ ozone concentration for 120 min with 435 mg L^−1^ RB 19.^52^ The biodegradability of treated RB 19 *via* the ozonation process was 0.33 and the TU value was below 1 since low molecular products were produced without presenting toxicity.^46^ RB 19 degradation was studied by the electro-Fenton process, and the results revealed that 87% TOC removal was achieved at pH 3 for 180 min. However, in this current work, both treatments by the ozonation membrane contactor and Fenton oxidation resulted in high COD removals compared to the study of Fanchiang and Tseng (2009)^46^ but less TOC removals than the studies of He *et al.* (2014)^53^ and Lovato *et al.* (2017)^52^.

**Table:** Comparison of RB 19 decolorization with the ozonation process and Fenton oxidation

Treatment	Treatment conditions	Performance	Ref.
Ozonation with sonolysis	[RB 19] = 500 mg L^−1^, [O_3_] = 3.8 g h^−1^, ultrasound density = 88 W L^−1^, pH = 8, temperature = 25 °C, time = 120 min	Pseudo-first-order rate (*k*) = 8.2 × 10^−3^ min^−1^	54
Ozonation process	[RB 19] = 100 mg L^−1^, volume = 4 L, [O_3_] = 88.8 mg min^−1^, pH = 5.7, time = 10 min	BOD_5_/COD = 0.33, COD removal = 36%, microtox toxicity < 1 (TU value)	46
Ozonation process	[RB 19] = 435 mg L^−1^, [O_3_] = 55.8 mg L^−1^, volume = 1 L, pH = 6, time = 120 min	RB 19 decolorization = 100%, TOC removal = 94%, inhibition of *V. fischeri* = 15%	52
Electro-Fenton process catalyzed by Fe_3_O_4_ nanoparticles (H_2_O_2_ generated *in situ*)	[RB 19] = 100 mg L^−1^, [Fe_3_O_4_] = 1.0 g L^−1^, volume = 200 mL, current density = 3.0 mA cm^−2^, pH = 3, temperature = 35 °C, time = 180 min	TOC removal = 87%, pseudo-first-order rate (*k*) = 3.21 × 10^−3^ min^−1^	53
Ozonation with membrane contactor	[RB 19] = 75 mg L^−1^, [O_3_] = 40 mg L^−1^, PVDF-PAM membrane, volume = 1 L, pH = 3, time = 90 min	RB 19 decolorization = 98.6%, COD removal = 63%, TOC removal = 14%, BOD_5_/COD = 0.21	Current work
Fenton process	[RB 19] = 75 mg L^−1^, [Fe^2+^] = 0.25 mM, [H_2_O_2_] = 1.5 mM, volume = 1 L, pH = 3, time = 90 min	RB 19 decolorization = 91.2%, COD removal = 71%, TOC removal = 12%, BOD_5_/COD = 0.14	
Photo-Fenton process	[RB 19] = 75 mg L^−1^, [Fe^2+^] = 0.25 mM, [H_2_O_2_] = 1.5 mM, volume = 1 L, light intensity = 7 W, pH = 3, time = 90 min	RB 19 decolorization = 82.6%, COD removal = 74%, TOC removal = 9%, BOD_5_/COD = 0.13	

### 3.5 Energy consideration

For comparing the different RB 19 decolorizations by ordinary Fenton, photo-Fenton, and ozonation membrane contactor, it is worth comparing the energy consumed by the treatments. Electrical energy (EE) can be calculated according to eqn (18):18
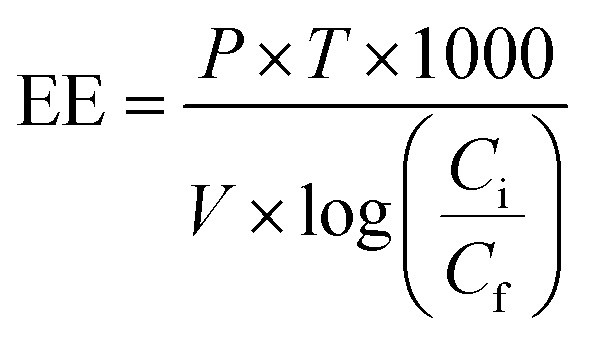
where *P* is the rated power (kW), *V* is the volume (L) of RB 19 water treated in time *T* (hour), and *C*_i_ and *C*_f_ are the initial and final concentrations of the target compound (mol L^−1^), respectively.

For the Fenton reaction, the energy corresponds to the magnetic stirrer plate (0.550 kW) during 1.5 h for 0.5 L of RB 19.19
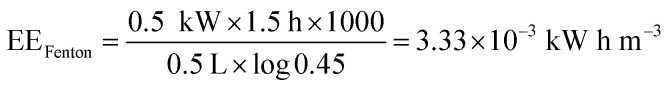


For the photo-Fenton reaction, the energy corresponds to the magnetic stirrer plate (0.550 kW) and UV light (0.007 kW) during 1.5 h.20
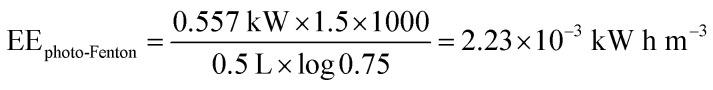


For the ozonation membrane contactor, the energy corresponds to the ozone generator and pump for the RB 19 flow rate. O_3_ was produced from 10 kWh kg^−1^ O_3_, the flow rate of O_3_ was 40 mg L^−1^ (4 × 10^−5^ kg L^−1^), and the gas flow rate was 0.6 L min^−1^ during 1.5 h. Therefore, the energy consumption of the ozone generator was 10 kW h kg^−1^ O_3_ × 4 × 10^−5^ kg L^−1^ × 0.6 L min^−1^ × 60 min h^−1^ = 0.144 kW. The energy consumption of the liquid pump was 3.47 × 10^−5^ kW.21
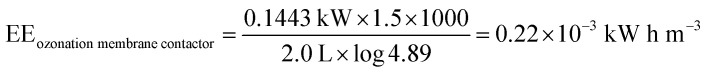


As can be seen, the ozonation membrane contacting process at 40 mg L^−1^ O_3_ achieved the highest RB 19 decolorization and required less electrical energy compared to the other techniques. This implied that RB 19 decolorization with ozonation based on the membrane contacting process was determined to present the lowest energy consumption as compared to those techniques based on Fenton oxidations. In general, the scale (full scale process and upscaling) and the quality of the water containing the scavenging agent have to be taken into account, as these could affect the decolorization and energy efficiency.

## 4. Conclusions

In this work, the decolorizations of RB 19 wastewater by the Fenton processes and ozonation with the membrane contacting process were studied under different experimental conditions. The optimum conditions for the decolorization of 75 mg L^−1^ RB 19 by the Fenton process were at pH 3, 1.5 mM H_2_O_2_ and 0.25 mM Fe^2+^ under room conditions. Under these conditions, 91.2% RB 19 decolorization efficiency was achieved within 90 min. The decolorization of RB 19 by the photo-Fenton process in the first 10 min was higher than that by the Fenton process, but after 90 min the decolorization efficiency of RB 19 by the Fenton process was higher than that by the photo-Fenton process. For ozonation with the membrane contacting process, the influence of affecting parameters on ozone flux, including gas velocity and liquid flow rate, was investigated. The results found that the ozone fluxes increased with gas velocities. The increase of liquid flow rates from 200 to 500 mL min^−1^ increased the ozone fluxes from 1.95 to 5.76 mg m^−2^ s^−1^. Moreover, the affecting parameters of decolorization efficiency, such as initial ozone and RB 19 concentrations and pH, were studied. The RB 19 decolorization efficiency increased from 99.0 to 99.6% when the ozone concentration increased from 30 to 50 mg L^−1^. The decolorization of RB 19 decreased when the initial RB 19 increased. The RB 19 decolorization efficiency at pH 3 was as high as that at pH 11. Therefore, the optimal conditions of the decolorization of RB 19 were found in the hollow membrane contactor with the ozonation process at 0.12 mM initial RB 19 concentration, 0.27 m s^−1^ ozone gas velocity, 400 mL min^−1^ RB 19 flow rate, 40 mg L^−1^ ozone concentration and at pH 3 and 11 for 90 minutes. The decolorization of RB 19 by ozonation with the membrane contactor using the PVDF-PAM membrane resulted in higher efficiency than by Fenton and photo-Fenton oxidations for 90 min. Also, the proposed ozonation with the membrane contactor using the PVDF-PAM membrane demonstrated significant anion mineralization of NO_3_^−^ and SO_4_^2−^ removals with high BOD_5_/COD but low energy consumption, indicating high competitiveness compared to Fenton and photo-Fenton oxidations. Therefore, the membrane contacting process is feasible and has potential for application in dye wastewater treatment.

## Conflicts of interest

There are no conflicts to declare.

## Supplementary Material

RA-011-D1RA01871J-s001
