# Supplementary material for: Efficient reactive blue 19 decolorization by the comparison of ozonation membrane contacting process and Fenton oxidation
Source: RSC Adv. 2021 May 17;11(29):17775–88. doi: 10.1039/d1ra01871j (PMC9033211; doi:10.1039/d1ra01871j)
Supplement: RA-011-D1RA01871J-s001 [file RA-011-D1RA01871J-s001.pdf]

Electronic Supplementary Material (ESI) for RSC Advances.
This journal is © The Royal Society of Chemistry 2021

Supplementary Material

Table S1 Ozone utilization with different gas and liquid velocities

**Gas velocity**		
**Gas velocity ($m\ s^{-1}$)**	**Ozone utilization ($mg\ L^{-1}$)**	**Flux of ozone ($mg\ m^{-2}s^{-1}$)**
0.13	10	1.33
0.27	10	2.66
0.40	8	3.19
0.53	7	3.72
**Liquid velocity**		
**Liquid flow rate ($mL\ min^{-1}$)**	**Ozone utilization ($mg\ L^{-1}$)**	**Flux of ozone ($mg\ m^{-2}s^{-1}$)**
200	11	1.95
300	12	3.19
400	13	4.61
500	13	5.76

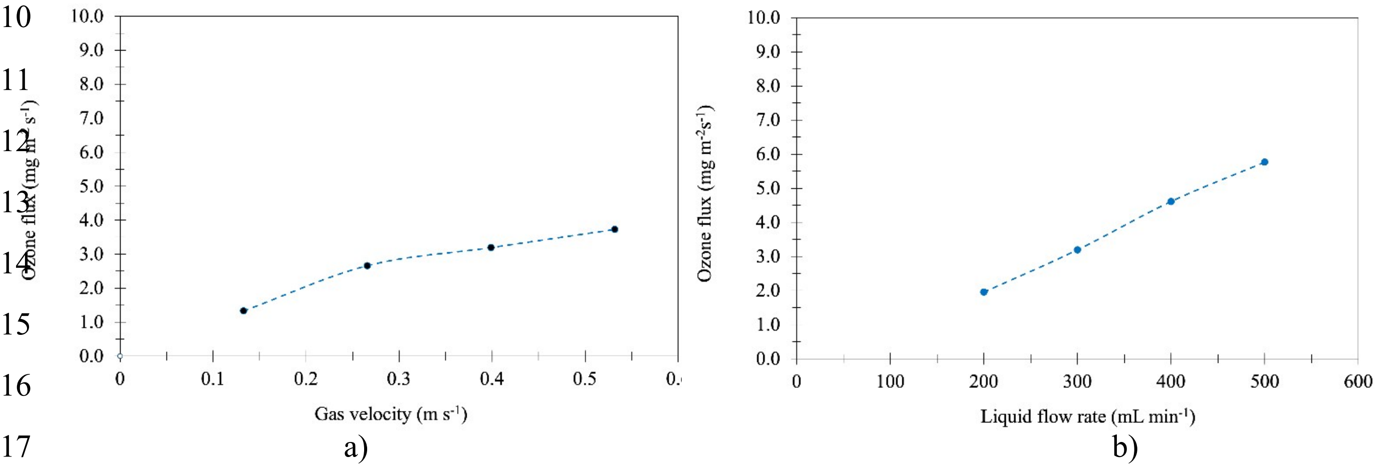

Figure S1. Effect of affecting parameters on ozone flux using 0.075 mM of RB 19: a) gas velocity and b) liquid velocity.

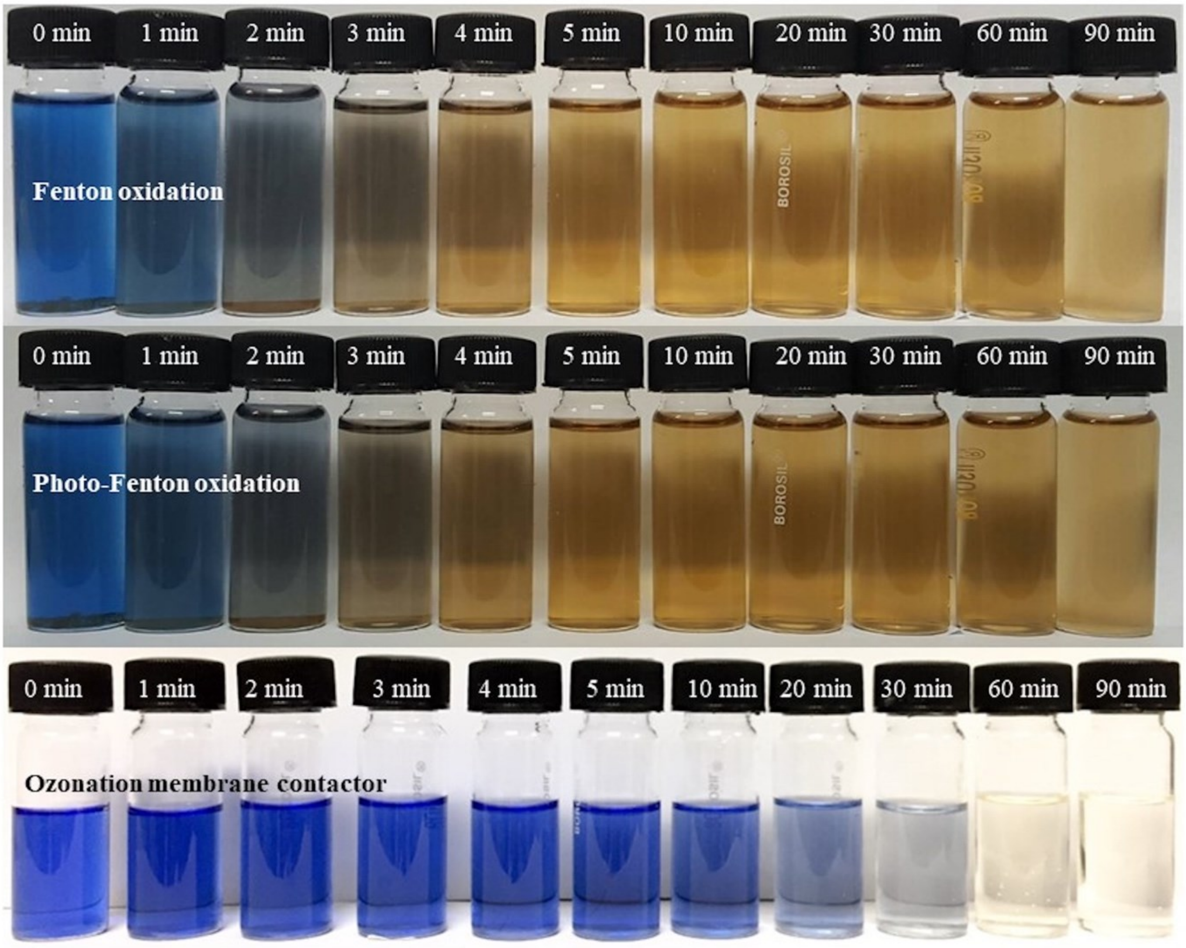

Figure S2. Illustration of RB 19 after treatment by Fenton oxidation, photo–Fenton oxidation and ozonation membrane contactor using PVDF–PAM membrane at different times.

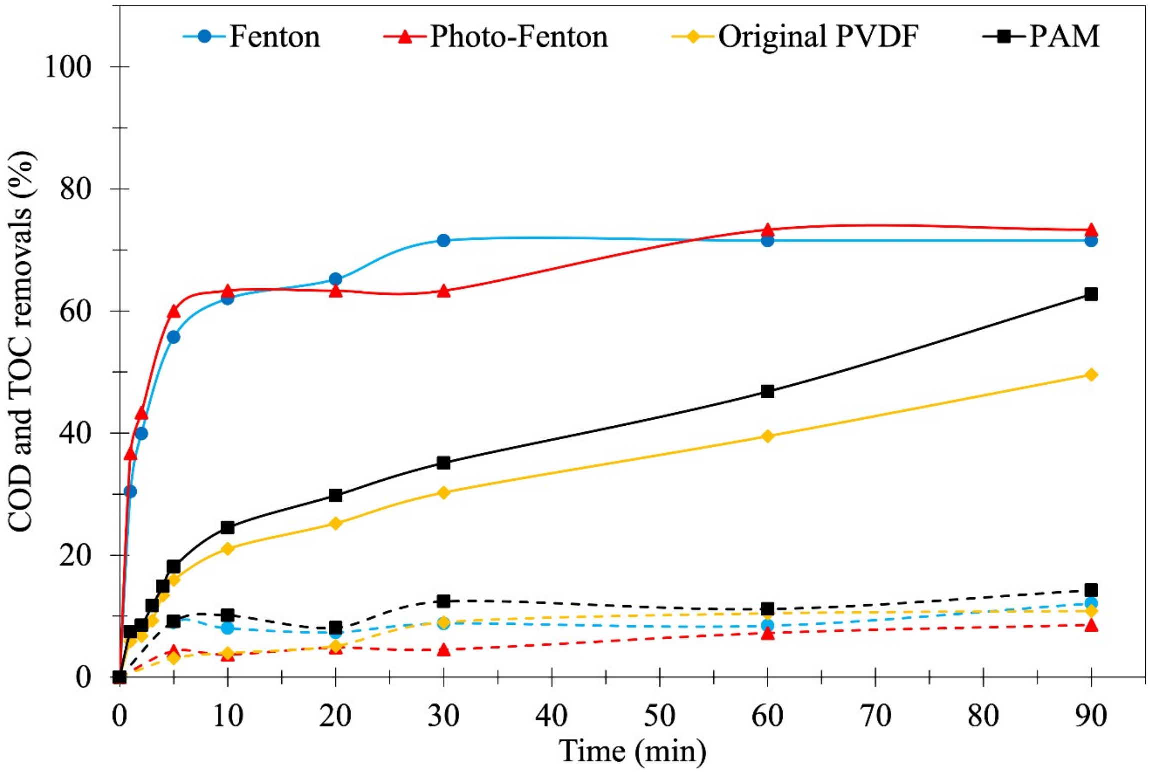

Figure S3. The variation of COD TOC and BOD_5/COD after 90 min of RB 19 decolorization by Fenton oxidations and ozonation membrane contactor with different membranes (solid lines = COD removal and dashed lines = TOC removal)
